# Outcomes in Dutch *DPP6* risk haplotype for familial idiopathic ventricular fibrillation: a focused update

**DOI:** 10.1007/s12471-023-01792-1

**Published:** 2023-07-27

**Authors:** Auke T. Bergeman, Wiert F. Hoeksema, Martijn H. van der Ree, Lucas V. A. Boersma, Sing-Chien Yap, Lisa M. Verheul, Rutger J. Hassink, Saskia N. van der Crabben, Paul G. A. Volders, Christian van der Werf, Arthur A. M. Wilde, Pieter G. Postema, European Reference Network for rare, low prevalence and complex diseases of the heart: ERN GUARD-Heart, Paul G. A. Volders, Christian van der Werf, Arthur A. M. Wilde, Pieter G. Postema

**Affiliations:** 1 Department of Cardiology, Amsterdam University Medical Centres, location Academic Medical Centre, University of Amsterdam, Amsterdam, The Netherlands; 2 Heart Failure and Arrhythmias, Amsterdam Cardiovascular Sciences, Amsterdam, The Netherlands; 3 Department of Cardiology, St. Antonius Hospital, Nieuwegein, The Netherlands; 4 Department of Cardiology, Erasmus University Medical Centre Rotterdam, Rotterdam, The Netherlands; 5 Department of Cardiology, Division Heart & Lungs, University Medical Centre Utrecht, Utrecht, The Netherlands; 6 Department of Human Genetics, Amsterdam University Medical Centres, location Academic Medical Centre, University of Amsterdam, Amsterdam, The Netherlands; 7 Department of Cardiology, Maastricht University Medical Centre, Maastricht, The Netherlands

**Keywords:** Sudden cardiac death, Idiopathic ventricular fibrillation, DPP6

## Abstract

**Background:**

The genetic risk haplotype *DPP6* has been linked to familial idiopathic ventricular fibrillation (IVF), but the associated long-term outcomes are unknown.

**Methods:**

DPP6 risk haplotype-positive family members (*DPP6* cases) and their risk haplotype-negative relatives (*DPP6* controls) were included. Clinical follow-up data were collected through March 2023. Implantable cardioverter-defibrillator (ICD) indication was divided in primary or secondary prevention. Cumulative survival and event rates were calculated.

**Results:**

We included 327 *DPP6* cases and 315 *DPP6* controls. Median follow-up time was 9 years (interquartile range: 4–12). Of the *DPP6* cases, 129 (39%) reached the composite endpoint of appropriate ICD shock, sudden cardiac arrest or death, at a median age of 45 years (range: 15–97). Median overall survival was 83 years and 87 years for *DPP6* cases and *DPP6* controls, respectively (*p* < 0.001). In *DPP6* cases, median overall survival was shorter for males (74 years) than females (85 years) (*p* < 0.001). Of the *DPP6* cases, 97 (30%) died, at a median age of 50 years. With a prophylactic ICD implantation advise based on risk haplotype, sex and age, 137 (42%) of *DPP6* cases received an ICD, for primary prevention (*n* = 109) or secondary prevention (*n* = 28). In the primary prevention subgroup, 10 patients experienced a total of 34 appropriate ICD shocks, and there were no deaths during follow-up. *DPP6* cases with a secondary prevention ICD experienced a total of 231 appropriate ICD shocks.

**Conclusion:**

Patients with the *DPP6 *risk haplotype, particularly males, are at an increased risk of IVF and sudden cardiac death. Using a risk stratification approach based on risk haplotype, sex and age, a substantial proportion of patients with a primary prevention ICD experienced appropriate ICD shocks, showing the benefit of prophylactic ICD implantation with this strategy.

## What’s new?

- Using contemporary risk stratification based on *DPP6* risk haplotype status, age and sex, a substantial proportion of patients with a primary prevention ICD carriers experienced appropriate implantable cardioverter-defibrillator (ICD) shocks.
- Estimates of median survival time in patients with the *DPP6* risk haplotype have increased substantially compared with earlier estimates, suggesting that initiation of cascade screening leading to early diagnosis and ICD treatment has reduced mortality.
- *DPP6* cases with a secondary prevention ICD represented a small but severely affected subset, that suffered from frequent IVF recurrences.

## Introduction

Survivors of sudden cardiac arrest (SCA) are diagnosed with idiopathic ventricular fibrillation (IVF) when no cause or other phenotypic expression (such as characteristic electrocardiographic or imaging features) can be identified after comprehensive assessment of structural and functional cardiac abnormalities [1]. Although ‘true’ IVF is rare, with studies reporting that 1.2–6.8% of SCA survivors with a shockable first rhythm could be diagnosed as such,[2, 3] it is an important public health concern, predominantly affecting young and otherwise healthy individuals.

Despite a family history of sudden cardiac death (SCD) in a substantial proportion of IVF patients,[4, 5] a genetic substrate is not (yet) identifiable in most patients. However, in the Netherlands, a founder risk haplotype (i.e. a series of base pairs that are similar for all carriers instead of a similar single variant or mutation) has been associated with SCD and familial IVF [6, 7]. This risk haplotype is located on chromosome 7q36 and comprises the gene for dipeptidyl aminopeptidase-like protein 6 (*DPP6*). In *DPP6* IVF, overexpression of *DPP6* appears to be critical to the phenotype [6]. As DPP6 is involved in the rapidly recovering cardiac transient outward potassium current (*I*_*to*_), it is thought that overexpression of this protein specifically increases the activity of Purkinje fiber *I*_*to*_, leading to a propensity for IVF [8].

*DPP6* risk haplotype-positive family members (hereafter referred to as ‘*DPP6* cases’), unlike their risk haplotype-negative relatives (‘*DPP6* controls’), have been extensively characterised. They are a group of patients who suffer SCD at young age from malignant short-coupled premature ventricular complexes originating predominantly from the lower part of the right ventricular free wall, without other clinical manifestations [9, 10]. *DPP6* IVF thus forms a subset of so-called short-coupled IVF [11]. Specifically, for risk stratification in patients who did not previously experience IVF, no clinically useful abnormalities are detected with electrocardiography or cardiac magnetic resonance imaging in affected patients [9]. Therefore, first-degree family members of diagnosed patients are offered presymptomatic genetic testing after genetic counselling.

Based on previous observations,[6, 9] recommendations for the treatment of *DPP6* cases have been formulated, of which the most pivotal aspect is the approach regarding implantable cardioverter-defibrillator (ICD) implantation. However, because of the lack of clinical features, risk stratification and management of *DPP6* cases remain challenging, particularly in asymptomatic patients. The aim of this study was to assess the long-term outcomes of the current cohort of *DPP6* cases and *DPP6* controls, thereby focusing on mortality, IVF recurrence and appropriate ICD therapy, including ICD complications.

## Methods

### Study population

The study population consisted of an extension of the previously described cohort of *DPP6* cases and their risk haplotype-negative relatives (*DPP6* controls)[9] and newly identified *DPP6* cases. The cohort of *DPP6* cases also included individuals with unexplained SCD at age < 50 years who could not undergo genetic analysis but were first-degree family member of a *DPP6* case (i.e. inferred or obligate carrier). *DPP6* cases with an ICD were divided by ICD indication as either primary prevention (when they did not previously experience IVF) or secondary prevention (when they previously survived IVF).

Clinical follow-up data on all-cause mortality, SCA, ICD status, quinidine use and the occurrence of appropriate and inappropriate shocks were collected from inception through March 2023. Vital status was obtained through linkage with the Dutch National Personal Records Database. ICD shocks were considered appropriate if they were delivered for ventricular tachyarrhythmias. To allow assessment of the yearly ventricular tachyarrhythmia/IVF recurrence, which is not skewed by VF storms, we used a definition of an arrhythmic event that comprised all appropriate ICD shocks within a 24-hour period. Informed consent for this type of data collection was obtained from all included patients.

### Statistical analysis

Continuous data are reported as mean ± standard deviation or median with interquartile range (IQR) and compared using the unpaired *t*-test or Mann-Whitney U test, depending on variable distribution. Categorical data are reported as number and percentage. Rates of (1) appropriate ICD shocks and (2) arrhythmic events were calculated by dividing the number of appropriate ICD shocks and arrhythmic events, respectively, by the corresponding number of patient-years. In addition, a composite endpoint of appropriate ICD shock, SCA or death was determined. Cumulative survival and event rates were compared with the log-rank test and are presented as survival curves. For survival analysis, follow-up was censored at the date of last follow-up. All reported *p*-values are two-sided and were considered statistically significant when < 0.05. All statistical analyses were performed using SPSS (version 28.0, IBM Corporation, Armonk, NY, USA).

## Results

### Baseline characteristics

In this study, 327 *DPP6* cases (175 (54%) male) and 315 *DPP6* controls (156 (50%) male) were included. Median follow-up duration was 9 years (IQR: 4–12; *n* = 180). Baseline characteristics of the included patients are shown in Tab. 1.

**Table 1:** Baseline characteristics of the study cohort

Characteristic	Total (*n* = 642)	*DPP6* cases (*n* = 327)	*DPP6 *controls (*n* = 315)
*Male*	331 (52)	175 (54)	156 (50)
*Age, years*	53 ± 19	51 ± 20	54 ± 18
*Deceased*	128 (20)	97 (30)	31 (10)
*Method of identification*			
DNA haplotype		260 (80)	309 (98)
Obligate carrier		42 (13)	6 (2)
SCD at age < 50 and first-degree family member of risk haplotype-positive patient		25 (8)	0 (0)

Data are *n* (%) or mean ± standard deviation

*SCD* sudden cardiac death

### Survival

**Fig. 1:**
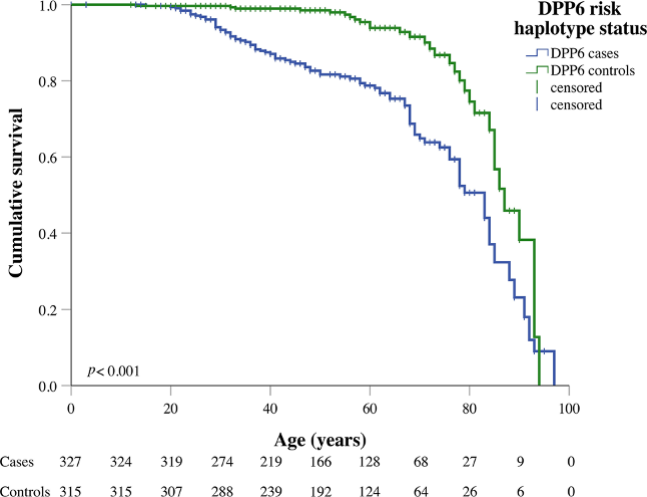
Survival curves showing mortality in *DPP6* cases and *DPP6* controls

Within the subgroup of *DPP6* cases, which included many family members who were not previously recognised as *DPP6* cases (i.e. obligate carriers), 97 (30%) died during follow-up (median age at death: 50 years), of whom 52 due to SCD. Of the *DPP6* controls, 31 (10%) died during follow-up (median age at death 73 years). Median survival significantly differed between *DPP6* cases and *DPP6* controls (83 vs 87 years; *p* < 0.001) (Fig. 1).

**Fig. 2:**
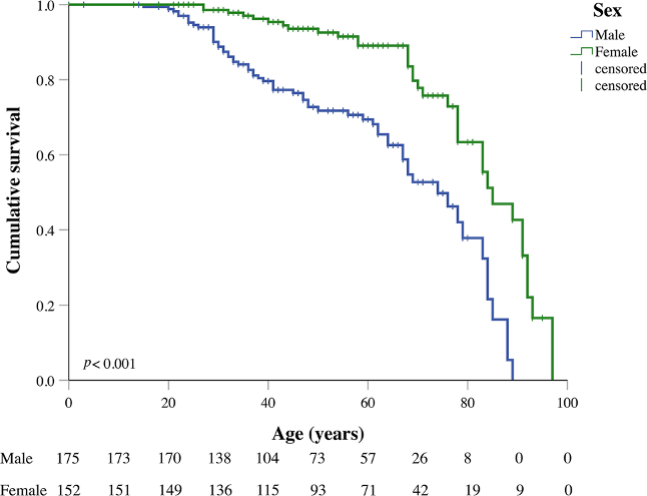
Survival curves showing mortality in male and female *DPP6* cases

Next, we analysed survival differences in *DPP6* cases by sex. Of the 97 deceased *DPP6* cases, 63 (65%) were male. Median survival time was 74 years and 85 years for males and females, respectively (*p* < 0.001) (Fig. 2).

**Fig. 3:**
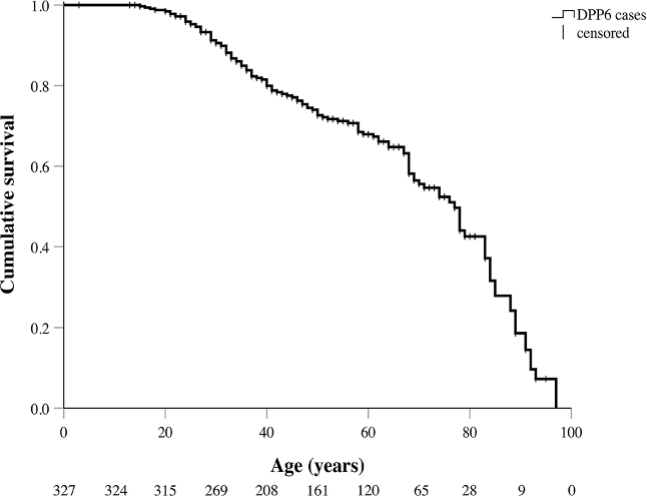
Survival curve showing mortality, aborted sudden cardiac arrest or first appropriate implantable cardioverter-defibrillator (*ICD*) shock in all *DPP6* cases

Among all *DPP6* cases, 129 (39%) reached the composite endpoint of appropriate ICD shock, SCA or death, at a median age of 45 years (IQR: 32–69) (Fig. 3). Four *DPP6* cases experienced an SCD or SCA before the age of 20 years: a 15-year-old male (SCD), a 16-year-old male (SCA) and two 17-year-old males (SCA). In the case of the 15-year-old, autopsy revealed findings compatible with (concurrent) hypertrophic cardiomyopathy. Within 1 year of ICD implantation for secondary prevention, all 3 SCA survivors experienced their first appropriate ICD shock. The youngest female *DPP6* case who died suddenly was 27 years old. The highest age at which SCD occurred was 68 years for females and 88 years for males.

### ICD outcomes

Of the 327 *DPP6* cases, 137 (42%) had an ICD implanted during follow-up. Reasons for not implanting an ICD were death before potential implantation/obligate carrier (*n* = 85), perceived low risk based on age (*n* = 74), patient refusal (*n* = 10) and unknown (*n* = 21). The median available follow-up duration after ICD implantation was 9 years (IQR: 5–13). Primary prevention of SCD was the most common indication (*n* = 109), followed by secondary prevention of SCD (*n* = 28). The ICD types were subcutaneous (*n* = 60), transvenous (*n* = 50), extravascular (*n* = 3), multiple at different moments (*n* = 7) and unspecified (*n* = 17).

In 6 patients, their ICD was explanted during follow-up (due to advanced age), of whom 5 received their ICD for primary prevention.

### Outcomes in primary prevention subgroup

The median follow-up duration in patients with a primary prevention ICD (*n* = 109) and those who (initially) refused ICD implantation for primary prevention (*n* = 10) was 8 years (IQR: 4–12). During this time, 10 patients (8%) experienced a total of 34 appropriate ICD shocks and there was 1 SCA (Tab. 2). The rate of appropriate ICD shocks and arrhythmic events per patient-year was 3.8 and 1.9%, respectively. None of the patients who received an ICD for primary prevention died, and there were no occurrences of aborted SCD. Of the patients with a primary prevention ICD, 9 took quinidine during follow-up, of whom 5 were still taking quinidine during the last visit. Of these 9 patients, 2 experienced IVF recurrence while being treated with quinidine (of which at least one had a subtherapeutic dose due to incompliance).

**Table 2:** Patient characteristics of 10 *DPP6* cases with indication for primary prevention ICD who experienced ≥ 1 appropriate shocks

ID	Age at diagnosis, years	Sex	Age at ICD implantation, years	Age at first appropriate ICD shock, years	Number of appropriate ICD shocks	Follow-up duration, years	Quinidine use
A	31	Male	31	41	2	14	Since first ICD shock
B	28	Female	28	40	1	12	Never
C	38	Male	38	46	2	14	Since second ICD shock
D	21	Female	21	32	4	14	Since third shock. Discontinued because of side effects
E	26	Male	26	37	9 (VF storm)	14	Since VF storm
F	14	Male	19	26	1	6	Never
G	40	Male	40	46	2	6	Since second ICD shock
H	36	Male	36	39	8	9	Never
I	40	Female	50^a^	51	2	14	Since second ICD shock
J	21	Male	21	26	3	5	Since third ICD shock

*VF* ventricular fibrillation

^a^Patient initially refused implantable cardioverter-defibrillator (*ICD*) implantation, until she experienced sudden cardiac arrest at age 50

### Outcomes in secondary prevention subgroup

The median follow-up duration after ICD implantation in the 28 patients with an ICD for secondary prevention, of whom 22 were males, was 12 years (IQR: 9–16). During the follow-up after ICD implantation, 21 patients (75%), of whom 16 males, experienced a total of 231 appropriate ICD shocks (median number of shocks per patients: 3; IQR 2–15). The yearly rate of appropriate ICD shocks was 70%. One patient who received an ICD for secondary prevention has since died. In this subgroup, 16 patients took quinidine during follow-up, of whom 15 were still on quinidine during the last visit.

### Quinidine

We identified 25 *DPP6* cases in the present cohort who were treated with quinidine. The indication for quinidine was almost always recurrent VF (*n* = 23); this included patients who initially received a primary prevention ICD. For the 2 other patients, the indication was paroxysmal atrial fibrillation (*n* = 1) or NSVT (*n* = 1). Seven patients experienced side effects. These were gastro-intestinal side effects (*n* = 4), dizziness (*n* = 1), defibrillation threshold elevation (*n* = 1) or unspecified (*n* = 1).

### ICD complications

Seventeen patients (12%) experienced ≥ 1 inappropriate shocks during follow-up, of whom 12 had a primary prevention ICD. Other ICD complications occurred in 19 patients: lead fracture (*n* = 7), ICD malfunction (*n* = 3), generator pocket infection (*n* = 2), pneumothorax (*n* = 2), lead dislocation (*n* = 1), endocarditis (*n* = 1), subclavian vein thrombosis (*n* = 1), generator pocket ulcer (*n* = 1) and lead perforation of the right ventricle (*n* = 1). None of these complications was lethal.

## Discussion

Patients with SCA in the absence of an identifiable cause present a challenging scenario for the clinician, as IVF is mostly defined by what is not known. As the first manifestation of IVF, including *DPP6* IVF, is typically SCA, and there are often no clinical symptoms or signs before such a devastating event, risk stratification is both crucial and difficult [12]. Moreover, IVF may present as a familial problem, further stressing the importance of adequate risk stratification in family members who did not (yet) experience IVF. This update of the long-term follow-up of the Dutch IVF *DPP6* risk haplotype cohort provides important new insights into the natural history of the condition and the value of ICD implantation using current risk stratification. Of additional significance is the size of this cohort, being among the largest founder cohorts worldwide [13, 14].

Our most consequential findings were derived from the patients with a primary prevention ICD, 8% of whom experienced appropriate ICD shocks during the substantial follow-up time. Most of the ICD shocks occurred around the fourth decade of life. Although there was a male predominance, 2 females were also severely affected. Although the arrhythmic event rate was relatively low in the primary prevention subgroup, it seemed to justify the current policy on ICD implantation, particularly when one considers that VF, as is characteristic in *DPP6* cases, would almost always lead to SCD in the absence of an ICD. Results from the subgroup with a secondary prevention ICD illustrated there was a small subset of severely affected individuals who suffered frequent IVF recurrences, often despite aggressive drug therapy. Regarding ICD complications, most of these complications were related to transvenous ICD leads.

Nannenberg et al. reported that estimates of survival duration in inherited arrhythmia syndromes appear to increase over time, as cascade screening allows for identification of family members with the genetic variant who may not have overt clinical symptoms [15]. For *DPP6* cases, it was demonstrated that after 2011, a ‘plateau’ was reached, where identification of more affected relatives no longer significantly affected survival estimates. Surprisingly, in the current study, we found that survival time increased substantially compared with these earlier estimates, suggesting that the initial overestimation of mortality in certain inherited arrhythmia syndromes may be even more marked. In addition, it seems reasonable to suggest that with initiation of cascade screening, family members at risk are now often diagnosed early and offered the option of ICD treatment, thereby reducing mortality.

Genetic analysis in survivors of SCA without a clearly identifiable cause enables identification of concealed arrhythmia syndromes and screening of relatives. Furthermore, the evaluation of specific genetic variants is an important aspect of risk stratification and management of patients with inherited arrhythmia syndromes [16]. In the absence of a clear phenotype, as is the case in IVF, determining which genes should be tested is complicated. The yield of genetic testing in these patients is relatively low, and variant classification is difficult [17, 18]. Based on a review of studies assessing the yield of genetic testing in IVF and recognising that a substantial proportion of patients with Brugada syndrome or long QT syndrome (LQTS) have a concealed phenotype, Visser et al. proposed a screening panel of *SCN5A, RYR2, CALM1* and the most common LQTS genes (*KCNQ1 *and* KCNH2*) [19]. If a (likely) pathogenic variant is identified, family screening may be indicated. In addition, there appear to be structural IVF subtypes, e.g. IVF with concomitant mitral annulus disjunction or mitral valve prolapse [20, 21].

At present, risk stratification in *DPP6* cases is based on the patient’s age and sex. For current clinical practice, we recommend clinical evaluation, genetic testing, and ultimately prophylactic ICD implantation in *DPP6* cases at the age of 16–17 years for males and 25–27 years for females. The recommended upper age limit of prophylactic ICD implantation is also a matter of continuous evaluation but is currently around 60 years for males and 65 years for females. After extensive discussions with our patients, we decided in some cases not to perform prophylactic implantation during a period of several years before these upper age limits.

We also recommend ICD explantation in asymptomatic *DPP6* cases at a sufficiently advanced age—i.e. around or above these upper limits, when the risk of VF episodes appears to be very low and equal to that of controls [9]. Although this study showed that SCD can occur at higher age, it is conceivable that common morbidities such as coronary artery disease are much more likely to contribute than previously asymptomatic *DPP6*, which is of course also a matter of continuous evaluation and risk-benefit assessments.

The efficacy of drug therapy in IVF, including quinidine, is another subject of investigation. For example, we documented a lethal VF storm in a *DPP6* patient who received amiodarone—possibly because amiodarone blocks the fast inward sodium current that would normally prevent unopposed *I*_to_ in phase 1 of the action potential. A similar observation in *DPP6* is that administration of ajmaline (e.g. to rule out Brugada syndrome in IVF patients who were not yet identified as *DPP6* carrier) may aggravate the phenotype and can result in short-coupled IVF (without signs of Brugada syndrome). This reaction to ajmaline has later also been documented in non-*DPP6* IVF [22].

## Conclusion

The management and risk stratification of familial IVF and *DPP6* cases remain challenging. Estimates of survival duration have increased since the first descriptions possibly due to active cascade screening and early therapy. Nonetheless, *DPP6* cases, particularly males, remained at an increased risk of mortality, and a substantial proportion of family members with a primary prevention ICD experienced appropriate ICD shocks, illustrating the benefit of prophylactic ICD implantation using a risk stratification approach based on risk haplotype, sex and age.
